# The Impact of High Complexity Total Pelvic Exenteration on Surgeon Fatigue: The FaME Study

**DOI:** 10.1111/ans.70362

**Published:** 2025-11-11

**Authors:** Miriam Khalil, Joshua R. Burke, Jim P. Tiernan, Aaron J. Quyn

**Affiliations:** ^1^ The John Goligher Colorectal Surgery Unit St James's University Hospital, Leeds Teaching Hospital Trust Leeds UK; ^2^ School of Medicine The University of Leeds Leeds UK

**Keywords:** dissection, fatigue, heart rate, monitoring, stamina, total pelvic exenteration

## Abstract

**Aims:**

Total pelvic exenteration surgery (TPE) is a radical procedure requiring significant surgeon mental and physical endurance. Literature surrounding surgeon fatigue remains minimal. This study investigated impact of performing high complexity (bony, vascular and neurological resection) TPE on surgeon fatigue.

**Methods:**

Two surgeons ([#1]/[#2]) delivering high complexity TPEs (1st November 2022–1st September 2023) in a tertiary centre (110/annum) were assessed using a predefined protocol. Surgeon heart rate, concentration performance (d2 Brickencamp test) and Surgery Task Load Index (SURG‐TLX) were measured across intraoperative stages (mobilisation, dissection, reconstruction). Surgeons underwent baseline cardiopulmonary exercise testing.

**Results:**

Baseline VO_2_ max and anaerobic thresholds: #1 = 37.6 mL/kg/min, 50%; #2 = 54.3 mL/kg/min, 70%. Mean error rate (ER): d2 test 1 (pre‐procedure) and 2 (post‐dissection): #1 = 3.58% [0.91%–8.60%] [SD = 3.16, 95% CI = 0.27 to 6.91], #2 = 4.46% [1.37%–7.23%] [SD = 2.55, 95% CI = 1.79 to 7.14]. Greater HR deviation correlated with higher test 2 ER for both subjects (*p* > 0.05). During dissection, HR increased from baseline: mean of 70% [#1] and 51.9% [#2]; max HR 106.7% [#1] and 111.5% [#2]. Subjects demonstrated smaller mean HR increase from baseline during two consultant dissection versus with trainees (#1 = 46.7% [*p* < 0.001]; #2 = 48.1% [*p* > 0.05]). Greater mean dissection time did not significantly affect mean test 2 ER (*p* > 0.05, 95% CI −2.88 to 4.90). SURG‐TLX situational stress domain was most impacted.

**Conclusions:**

Surgeon HR fluctuations corresponded with significant intraoperative events. Assistant dependent HR fluctuation and increased average d2 ER was demonstrated by greater HR deviations during dissection. Further investigation optimising strategies minimising surgeon fatigue is required.

## 1 Introduction

Total pelvic exenteration (TPE) surgery is a complex, radical procedure, which in males involves the removal of the rectum, prostate, lower colon, and bladder; in female patients, the vagina, uterus, ovaries, and bladder are removed [1]. Following anterior and posterior compartment resection during TPE, a urostomy and colostomy are formed [2]. High complexity TPE encompasses conventional TPE, involving *en bloc* resection of pelvic viscera, with extension to remove bony structures including the sacrum with or without pubic bones, and structures within the pelvic sidewall including major vessels and sciatic nerves [3]. Pelvic exenteration surgery is resource intense, whilst being challenging both physically and technically for the surgical team. However, its evolution during the past decade has afforded patients with advanced tumours the opportunity for cure where they would previously have been considered palliative [4]. Understandably, this extended surgical procedure is associated with high morbidity; benchmark outcomes for patients undergoing pelvic exenteration for locally advanced primary (LARC) and recurrent rectal cancer (LRRC) demonstrate the major complication rate calculated benchmark threshold of ≤ 44% for LARC and ≤ 53% for LRRC respectively [5]. Appropriately, the current literature focus has been centred around patient outcomes as the procedure has evolved to become more radical. However, the toll on the operating surgeon and possible impact on performance remains poorly understood.

Muscular and non‐muscular fatigue inevitably occur when performing complex surgical procedures [6], with lengthy procedures further contributing to operative fatigue. Surgeons performing high complexity pelvic exenteration surgery are required to practise significant focus, mental stamina, and attention to detail for prolonged time periods. Together with high cognitive demands are the significant physical demands sustained by exenteration surgeons [7].

Alongside the increasing complexity of exenterative procedures, increased indications for the surgery have contributed to increased workloads for these surgeons. When considering the multitude of factors contributing to burnout, increasing workloads are at the forefront. Burnout can be defined as a declining state that can impact both an individual's personal and professional life [8, 9], it can manifest as behavioural and/or mental characteristics, including emotional exhaustion, depersonalisation, or a feeling of lack of personal accomplishment [10, 11]. Overall surgeon burnout rate in the UK is reported at 32% [8], with the highest rates reported by Robinson et al. in surgical trainees of varying specialties and grades at 59% [12] compared to a consultant average of 41.0% [8, 11, 13]. Interestingly, the highest levels of emotional exhaustion in several studies have been reported in consultants, at 41.0% in surgical oncology [13], and 31.7% in colorectal and vascular surgeons [14]. Burnout remains one of the core drivers contributing to declining mental health in some of the medical community. In the context of increasing surgical complexity, it is crucial that we can combine the delivery of high‐quality surgery whilst protecting our surgeons from burnout. The multiple factors possibly influencing surgical fatigue should be investigated further with the aim of understanding how solutions reducing this fatigue could be incentivised and implemented.

The study aims to examine the impact of performing high complexity TPE on surgeons' individual mental and physical fatigue. The overarching study aim focuses on improving awareness amongst clinicians regarding the impact of surgical fatigue during complex surgical procedures. The findings from this study could result in widespread recognition across all surgical disciplines performing extended surgery, with the aim of strengthening procedural changes in high complexity surgery.

## 2 Methods

### 2.1 Study Design

Between November 2022 and August 2023, both #1 and #2 had fatigue monitored during high complexity TPE. A total of 24 patients were included during this period. All statistical testing utilised two‐sided 5% significance levels, performed in IBM SPSS Statistics 28. Results have been reported as point estimates, alongside 95% confidence intervals and *p*‐values where applicable. This is a prospective cohort study evaluating the relationship between high complexity TPE surgery, procedure duration, and its impact on surgeon fatigue. Data release and ethical considerations were discussed and approved with participants and the University of Leeds Ethics Committee (Reference MREC 22–010). Variables examined will be through monitoring participants. Both response and non‐response bias will be minimised through this design [15].

### 2.2 Outcome Measures

Three objective parameters: (1) Cardiopulmonary exercise testing (CPET), (2) Heart rate, (3) d2 Test of Attention [16], were used to determine the primary outcome of performing high complexity TPE on surgeons' individual mental and physical fatigue. The unpredictability of TPE surgery forecasted has meant standardised procedural points agreed by participants prior to data collection will allow consistent windows for completion of d2 tests of attention. The SURG‐TLX (Surgery Task Load Index), a validated questionnaire, was completed prior to and following surgery. This is a multi‐dimensional surgery‐specific workload measure examining task complexity, distractions, situational stress, alongside mental, physical, and temporal demands. CPET was performed on participants prior to data collection, extrapolating VO_2_ max and anaerobic/lactate thresholds to ascertain underlying cardiopulmonary function. Surgeon heart rate will be measured throughout each TPE by monitoring the posterior tibial pulse through wearable devices. The study aims to identify and correlate heart rate spikes away from baseline heart rate to high‐risk intraoperative steps. Formal permission has been granted by Hogrefe Publishing to utilise the Brickencamp d2 test of attention, a validated cognition test measuring attention to detail and concentration performance.

### 2.3 Selection of Study Participants

Any surgeon carrying out a TPE during the study period was considered from a tertiary centre delivering a high volume of high complexity TPE (110 per annum). In this centre, two exenteration surgeons selected for participation perform the oncological resection without involving other specialist teams of gynaecology, urology, orthopaedics, or vascular surgery and perform the urological, gastrointestinal, and abdominal wall reconstruction themselves. Plastic surgery performs perineal reconstruction when required. Informed consent was gained by both participants, with selection aiming to show the impact of extreme surgery on such surgeons.

### 2.4 Ethics

Guidance by The University of Leeds ethical standards was sought and approved by the chair of the research ethics committee (Reference MREC 22‐010) whereby full ethical approval for this study design was not required. The study design and data produced align with the University of Leeds ethical standards in terms of participants' rights, ensuring informed consent, appropriate confidentiality, anonymising data, and creating an environment where concerns can be clearly raised and communicated.

### 2.5 Data Collection

This study analyses data from 24 high‐complexity TPE surgeries (*n* = 12 for each participant) during a total study period of 40 weeks. Three distinct TPE stages were defined as follows: (1) Mobilisation: mobilisation of structures along normal surgical planes, including colonic, ureter to pelvic brim, and bladder; (2) Dissection: identification and ligation of vascular pedicles, sacral nerves, and ligaments; (3) Reconstruction: formation of anastomoses including ileal conduit and perineal reconstruction. Only high‐complexity TPEs were eligible for inclusion during this study, with all conventional TPEs being excluded from data analysis.

## 3 Results

### 3.1 Baseline Cardiopulmonary Exercise Testing (CPET)

Both #1 and #2 were subject to CPET using mechanically braked cycle ergometers (Watts) during October 2022 at The University of Leeds, Sports and Exercise Medicine Testing Centre. Both participants opted for maximal exercising testing. No cardiac, respiratory, or general co‐morbidities were identified. Neither surgeon was taking medication during the study period. Baseline VO_2_ max and anaerobic/lactate thresholds: #1 = 37.6 mL/kg/min (2.9086 L/min), 50% and #2 = 54.3 mL/kg/min (4.7254 L/min), 70% respectively. Watts and maximum HR at anaerobic thresholds: #1 = 154.2 W, maximum HR = 115 bpm and #2 = 305.8 W, maximum HR = 146 bpm.

### 3.2 Heart Rate

**FIGURE 1:**
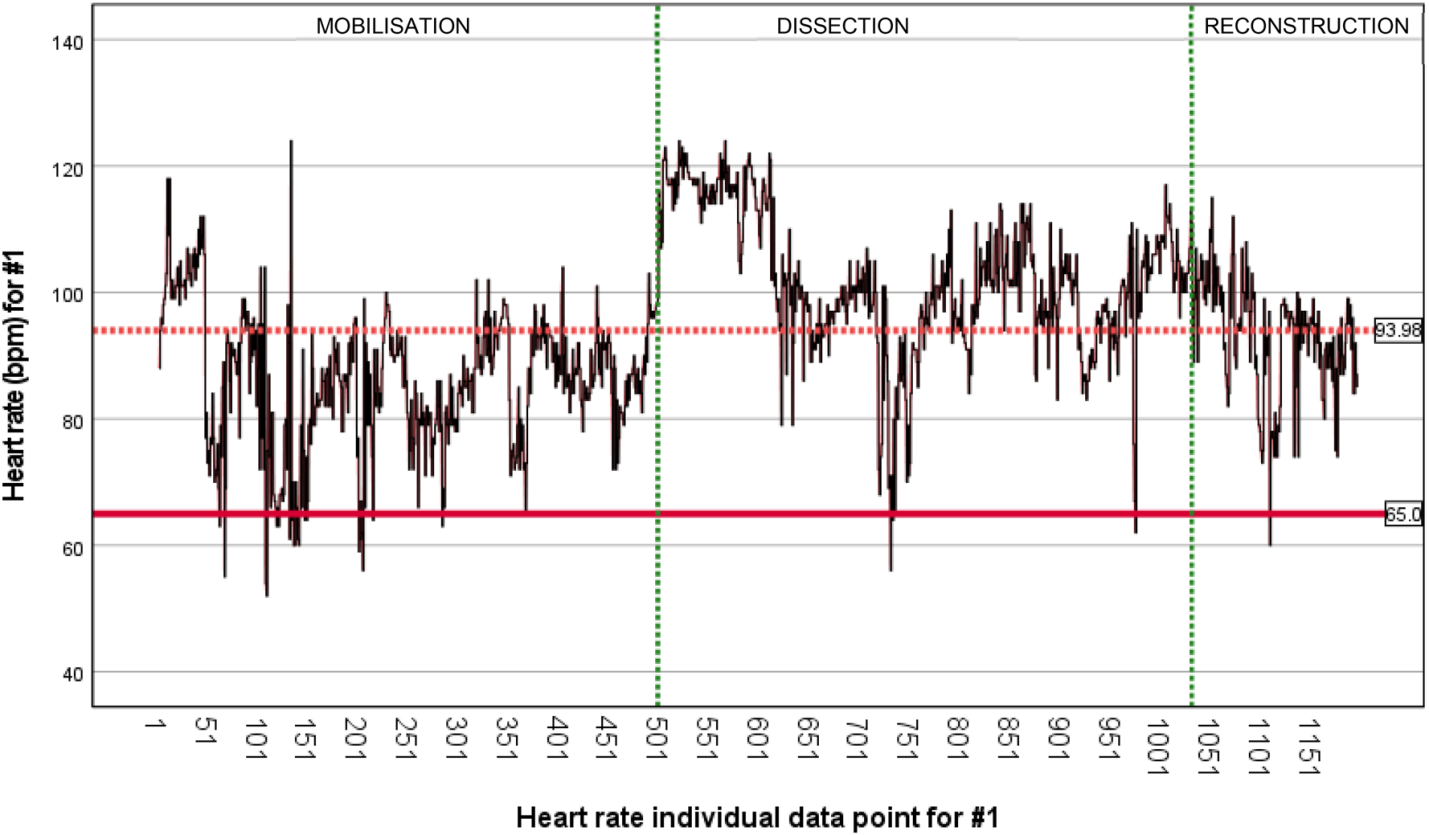
Heart rate across three operative stages of TPE for #1.

**FIGURE 2:**
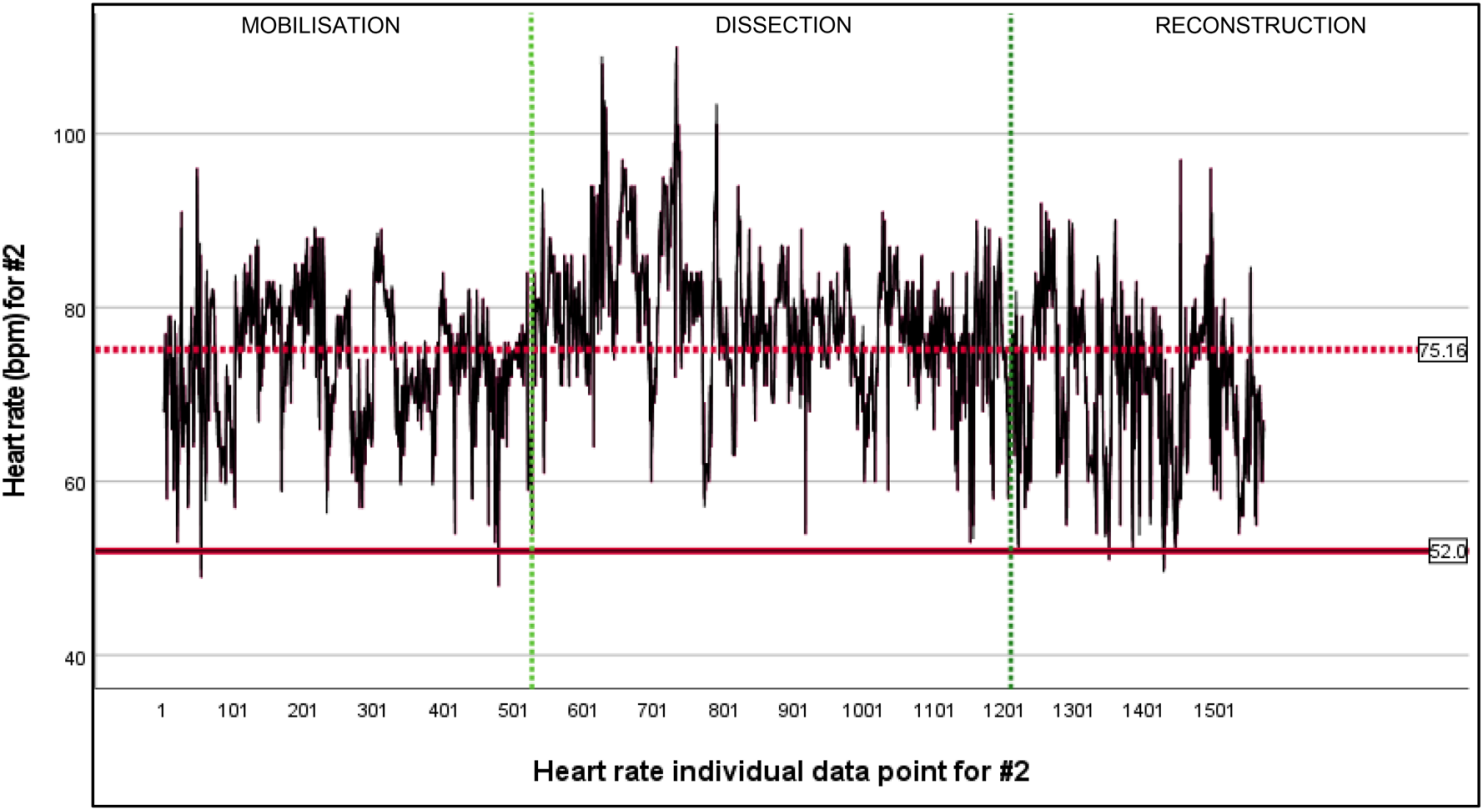
Heart rate across three operative stages of TPE for #2.

Both subjects were present for the entirety of TPE, defined as from the first incision to closure following the reconstruction phase. Heart rate (HR) data for #1 and #2 were normally distributed as assessed by both Kolmogorov–Smirnov (*p* < 0.001) and Shapiro–Wilk (*p* < 0.001) tests (Tables S1.1 and S1.2). Individual HR data points for #1: *n* = 1195, and #2: *n* = 1570. Mean HR beats per minute (bpm) across all three operative stages (mobilisation, dissection, reconstruction) were #1 = 93.98 bpm (baseline resting HR = 65 bpm) (Figure 1) [SD = 13.008] (Table S2.1) and #2 = 75.16 bpm (baseline resting HR = 52 bpm) (Figure 2) [SD = 8.805] (Table S2.2). Mean HR bpm during dissection for #1 = 107.65 bpm [SD = 10.366] and #2 = 83.12 bpm [SD = 7.787]. During TPE dissection, mean HR for both #1 and #2 increased from baseline by a mean of 70% [#1] and 51.9% [#2] with a max HR of 106.7% [#1] and 111.5% [#2]. A paired‐samples t‐test was used to determine if there was a significant difference in #1 and #2 mean HR during TPE dissection when operating together versus with a junior trainee. Both #1 and #2 demonstrated a reduced increase in mean HR from baseline during TPE dissection when operating together versus with junior trainees (#1 = 46.7% [*p <* 0.001] and #2 = 48.1% [*p >* 0.05]).

### 3.3 d2 Test of Attention

**FIGURE 3:**
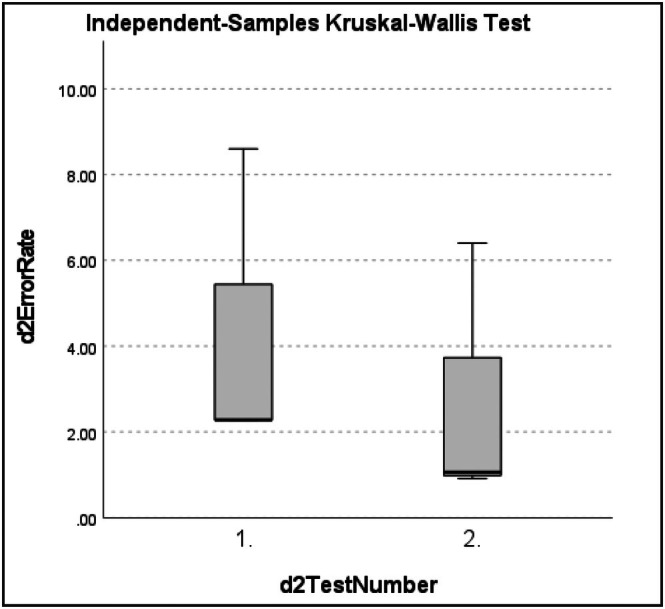
Independent samples Kruskal–Wallis test for #1 d2 test of attention error rate.

**FIGURE 4:**
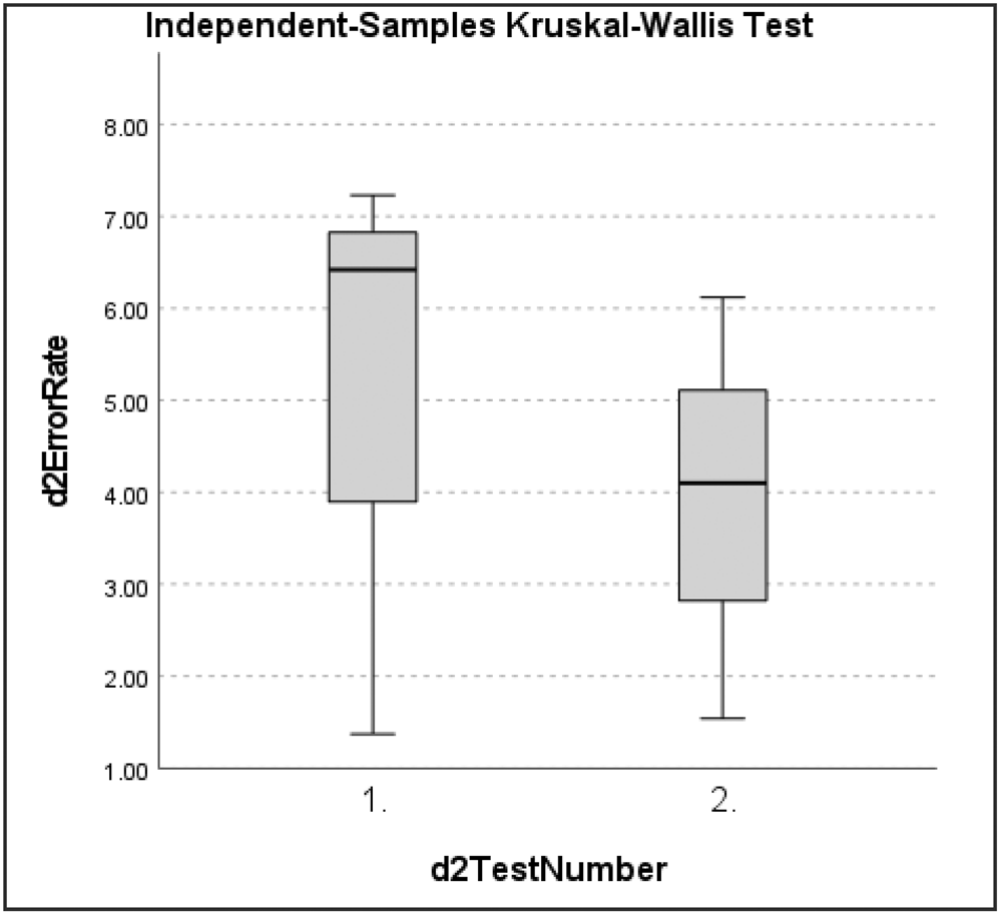
Independent samples Kruskal–Wallis test for #2 d2 test of attention error rate.

d2 error rate (ER) data for both #1 and #2 were non‐normally distributed as assessed by the Shapiro–Wilk test (*p >* 0.05) (Tables S3.1 and S3.2). Mean error rate (ER): d2 test number 1 and 2: #1 = 3.58% [0.91%–8.60%] [SD = 3.16, 95% CI = 0.27 to 6.91] (Table S4.1), #2 = 4.46% [1.37%–7.23%] [SD = 2.55, 95% CI = 1.79 to 7.14] (Table S4.2). Mean ER between d2 test 1 (pre‐procedure) and test 2 (post dissection period) decreased in both #1 = −1.60% and #2 = −1.09%, but these did not reach statistical significance for either subject. Independent samples Kruskal–Wallis test: (#1: H(1) = 1.190, *p =* 0.275 (Table S5.1) and #2: H(1) = 0.429, *p =* 0.513) (Table S5.2) between d2 ER for test number 1 and test 2 for both subjects (Figures 3 and 4).

### 3.4 Heart Rate and d2 Test of Attention Error Rate

Simple linear regression was calculated to examine whether mean heart rate increase from baseline demonstrated by both #1 and #2 during the TPE dissection period significantly predicted post‐dissection d2 test ER. The fitted regression model was (#1: mean HR bpm [TPE dissection period] 104.174 bpm + 1.969% (mean d2 ER test 2) and #2: 91.122 BPM + −2.327), respectively. The overall regression was not statistically significant (#1: *R* ^2^ = 0.363, F(75.821, 132.845) = 0.571, *p* > 0.05 and #2: *R* ^2^ = 0.549, F(57.060, 49.940) = 1.216, *p >* 0.05).

### 3.5 Operation Time and d2 Test of Attention Error Rate

An unpaired samples t‐test was used to determine whether an increased mean dissection period time for both subjects had a significant difference on mean d2 test post‐dissection period error rate. Operation total time for #1 ranged from 9.7 h to 14.62 h (SD 2.49) and #2 11.68–14.02 h (SD 1.29). Mean dissection time and mean d2 test post‐dissection error rate for subject #1 was 5.68 h [2.80 h–11.18 h, SD 4.77] and 2.82% [0.91%–6.40%], SD 3.13; for subject #2 it was 3.06 h [2.05 h–4.60 h, SD 1.36] and 2.96% [1.54%–6.12%, SD 2.30]. For both subjects, mean dissection period time during high complexity TPEs did not significantly affect mean d2 test post‐dissection period error rate (*p >* 0.05, 95% CI −2.88 to 4.90).

### 3.6 SURG‐TLX

The SURG‐TLX (a multidimensional surgery‐specific workload measure) assessed six dimensions: task complexity, distractions, situational stress, in addition to mental, temporal, and physical demands. Both surgeons reported the situational stress domain was most impacted.

## 4 Discussion and Conclusions

During dissection, mean HR increased markedly from baseline for both subjects: 70% [#1] and 51.9% [#2], with a maximum HR increase of 106.7% [#1] and 111.5% [#2], respectively. Smaller mean HR increase from baseline during dissection was demonstrated when both consultants operated together versus with junior trainees: #1 = 46.7% [*p* < 0.001] and #2 = 48.1% [*p* > 0.05]. Greater mean dissection time did not significantly affect mean test 2 (post dissection) error rate, *p* > 0.05, 95% CI −2.88 to 4.90.

In this study, we report the impact of performing high complexity TPE surgery on surgeon mental and physical fatigue at a high‐volume academic tertiary centre in the UK. Our main findings demonstrate that spikes in surgeon heart rate correlate to significant intraoperative events, alongside the TPE dissection period influencing an increase in surgeon heart rate from baseline most significantly. We illustrate that there is an assistant‐dependent influence on surgeon heart rate, with greater increases shown in mean heart rate when senior surgeons were operating with junior trainees compared with each other. Our results demonstrate that an increase in mean heart rate from baseline during TPE dissection did not significantly impact mental fatigue. We demonstrate that overall operation duration and mean dissection period duration do not significantly affect mental fatigue as examined through the d2 test of attention for detail. Our results demonstrate a positive finding that despite significant increases in surgeon heart rate during the dissection period of TPE, surgeons were able to sustain significant focus on mental tasks, irrespective of operation duration and complexity.

Understandably, literature for this radical procedure frequently focuses on clinical, oncological, and quality of life outcomes of patients due to the radicality of enduring such a major surgery. There is a paucity in the literature of studies monitoring and quantifying surgeon fatigue in general, with a complete literature gap on measuring surgeon fatigue during TPE; we are confident that our study findings are the first of their kind for pelvic exenteration surgeons specifically. Mazzella et al. investigated two surgeons' autonomic nervous system (ANS) and physiological responses to stress during thoracic surgery. This study reported lower maximum, minimum, and mean heart rate, lower respiratory frequencies, body temperatures, and lower mean desaturations of the two surgeons when performing robotic versus an open approach for anatomical lung resections. Although this study reports advanced measuring of physiological responses to stress surgeons endure during surgery, no quantifiable impact on mental fatigue was demonstrated alongside [17]. We appreciate that the application of measuring such parameters alongside would be interesting to apply to surgeons performing TPE, which is classed as a high fatigue procedure.

One of the clear objectives of our study was to investigate whether increased mean heart rate during the TPE dissection period predicted worse concentration and attention to detail as measured by the error rate of the d2 cognitive tests. Our data concluded there is no significant ER difference (*p >* 0.05) between test 1 (pre‐procedure) and test 2 (post dissection period) ER for both surgeons and therefore failed to reject the null hypothesis. Quantifying the impact of fatigue on surgeon neurophysiologic measures was demonstrated by Kahol et al., showing a significant (*p <* 0.014) decrease in surgical proficiency through increased cognitive errors on simulation tasks post on‐call, in addition to a 51% increase in workload score through wearing of an EEG‐cap [18]. Our results similarly demonstrated an increase in workload through the SURG‐TLX findings of both surgeons reporting the situational stress domain of the surgery task load index workload measure being most impacted following TPE.

Our findings align with the growing body of literature evidencing the accumulative toll of physically and mentally demanding surgery on surgeon wellbeing. A recent systematic review highlights the widespread incidence of WRMSK injuries across studies ranging from 66% to 94%, especially in open and laparoscopic surgery [7], with neck pain alone being reported in up to 87% of surgeons [19]. Fatigue should therefore not be viewed in isolation, as it often co‐exists with musculoskeletal strain. It is therefore not only this physical discomfort, but also surgeons experiencing mental apprehension in the forms of feelings of burnout and fear of career longevity loss due to physical pain from WRMSK injuries [7].

Whilst non‐muscular fatigue has been incontrovertibly correlated to reduced job efficiency and increased error incidence in other professions such as the aviation industry, the subject of the impact of fatigue on surgical performance remains controversial [20, 21, 22]. For example, it was found that 50% of surgeons' waking hours could be spent in this fatigued state, highlighting its impact on surgeon performance [23]. Contrasting this, Schlosser et al. reported improved cognitive performance on high‐fidelity virtual reality simulator tasks of surgical residents following reduced sleeping hours and alertness in a post on‐call state. The results of the d2 test in this study also illustrated improved objective alertness of surgeons following the stressor, ours being TPE and this being a post on‐call state. Similarly, an improved learning effect as opposed to improved mental alertness following a fatigue‐inducing variable might account for this improvement [24]. We also hypothesise that these objective improvements with a lowered d2 error rate post‐dissection period when compared to pre‐procedure test resulted from this learning effect, combined with subjective coping strategies and acquisition of high levels of subjective alertness practiced by senior surgeons.

This study monitored two pelvic exenteration specialist surgeons at a high‐volume UK exenteration unit. We appreciate the relatively small number of exenteration surgeons worldwide and recognise that selection of all surgeons eligible in this centre for monitoring translated to participants being *n =* 2, meaning selection bias is likely to exist in our cohort. Collection of data was impacted by industrial action limiting the TPE procedure number forecasted over the 11‐month period. Both participants aimed to complete all d2 tests at standardised TPE operative stages; understandably, such extreme surgery is unpredictable, and some procedure days did not allow for full completion of d2 tests, which limited this data set.

The evolving nature of pelvic exenteration surgery has meant patients with pelvic malignancy once deemed unresectable now frequently are offered this surgery with curative intent. Significant improvements in patient outcomes have been achieved over this time period, but investigation of the impact on surgeons performing high complexity TPE was absent prior to this study. We understand monitoring fatigue is a challenging and complex concept but remain confident that this study positively highlights that despite demonstrable increases in mean heart rate for both participants throughout TPE, it did not negatively impact either surgeons' concentration or performance on mental tasks.

### 4.1 Future Work

Although these data and study methods represent the first of their kind with regard to monitoring surgeon fatigue in pelvic exenteration surgery, we recognise that the data analysed is still relatively small in terms of numbers. This study aims to expand and explore our understanding of the impact of operative fatigue on surgical outcomes and surgeon longevity, and we recognise the future need to explore surgeon fatigue in tandem with physical ergonomic strain to safeguard surgeon well‐being. We highlight the impact of a high‐fatigue procedure on surgeons, with future work aimed at disseminating study concepts to monitor surgeons during more heterogeneous surgeries. We aim to study stress and fatigue responses objectively, highlighting any differences between female and male surgeons performing exenterative surgery. As common risk factors identified for burnout include younger surgeon age and clinical grade, we specifically aim to apply study concepts to surgeons at a junior stage of their training, with the aim of improving self‐awareness of fatigue and the application of coping strategies for fatigue. This study emphasises the need to further investigate factors influencing surgeon fatigue and the implementation of strategies to minimise this most effectively.

## Ethics Statement

This study design and data produced did not require full ethical approval by the University of Leeds research ethics committee (Reference MREC 22‐010).

## Consent

The authors have nothing to report.

## Conflicts of Interest

The authors declare no conflicts of interest.

## Supporting information


**Table S1.1.** Tests of normality for #1 heart rate data.
**Table S1.2.** Tests of normality for #2 heart rate data.
**Table S2.1.** Descriptives of heart rate data for #1.
**Table S2.2.** Descriptives of heart rate data for #2.
**Table S3.1.** Tests of normality for #1 d2 error rate.
**Table S3.2.** Tests of normality for #2 d2 error rate.
**Table S4.1.** d2 Error rate descriptives for #1.
**Table S4.2.** d2 Error rate descriptives for #2.
**Table S5.1.** Independent samples Kruskal–Wallis test for #1 showing d2 ER between test 1 and 2.
**Table S5.2.** Independent samples Kruskal–Wallis test for #2 showing d2 ER between test 1 and 2.

## Data Availability

The data that support the findings of this study are available from the corresponding author upon reasonable request.
