# Supplementary material for: The Impact of High Complexity Total Pelvic Exenteration on Surgeon Fatigue: The FaME Study
Source: ANZ J Surg. 2025 Nov 11;95(12):2518–24. doi: 10.1111/ans.70362 (PMC12717490; doi:10.1111/ans.70362)
Supplement: Supplementary file 1 — Table S1.1. Tests of normality for #1 heart rate data. Table S1.2. Tests of normality for #2 heart rate data. Table S2.1. Descriptives of heart rate data for #1. Table S2.2. Descriptives of heart rate data for #2. Table S3.1. Tests of normality for #1 d2 error rate. Table S3.2. Tests of normality for #2 d2 error rate. Table S4.1. d2 Error rate descriptives for #1. Table S4.2. d2 Error rate descriptives for #2. Table S5.1. Independent samples Kruskal–Wallis test for #1 showing d2 ER between test 1 and 2. Table S5.2. Independent samples Kruskal–Wallis test for #2 showing d2 ER between test 1 and 2. [file ANS-95-2518-s001.docx]

**Supplementary Table S1.1.** Tests of normality for #1 heart rate data.

| **Tests of Normality** | | | | | | |
| --- | --- | --- | --- | --- | --- | --- |
|  | Kolmogorov-Smirnov^a^ | | | Shapiro-Wilk | | |
|  | Statistic | df | Sig. | Statistic | df | Sig. |
| Heartratebpm#1 | .041 | 1195 | <.001 | .991 | 1195 | <.001 |
| a. Lilliefors Significance Correction | | | | | | |

**Supplementary Table S1.2.** Tests of normality for #2 heart rate data.

| **Tests of Normality** | | | | | | |
| --- | --- | --- | --- | --- | --- | --- |
|  | Kolmogorov-Smirnov^a^ | | | Shapiro-Wilk | | |
|  | Statistic | df | Sig. | Statistic | df | Sig. |
| Heartratebpm#2 | .053 | 1570 | <.001 | .991 | 1570 | <.001 |
| a. Lilliefors Significance Correction | | | | | | |


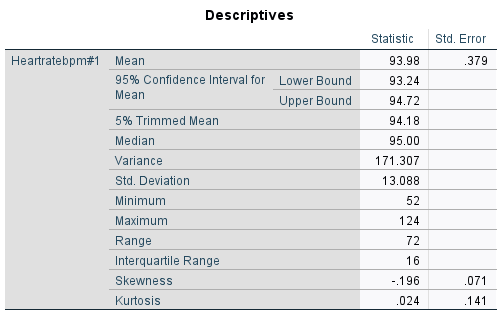
**Supplementary Table S2.1** Descriptives of heart rate data for #1.

**Supplementary Table S2.2.** Descriptives of heart rate data for #2.

| **Descriptives** | | | | |
| --- | --- | --- | --- | --- |
|  | | | Statistic | Std. Error |
| Heartratebpm#2 | Mean | | 75.16 | .222 |
|  | 95% Confidence Interval for Mean | Lower Bound | 74.73 |  |
|  |  | Upper Bound | 75.60 |  |
|  | 5% Trimmed Mean | | 75.16 |  |
|  | Median | | 76.00 |  |
|  | Variance | | 77.521 |  |
|  | Std. Deviation | | 8.805 |  |
|  | Minimum | | 48 |  |
|  | Maximum | | 110 |  |
|  | Range | | 62 |  |
|  | Interquartile Range | | 11 |  |
|  | Skewness | | -.072 | .062 |
|  | Kurtosis | | .392 | .123 |

**Supplementary Table S3.1** Tests of normality for #1 d2 error rate.

| **Tests of Normality** | | | | | | |
| --- | --- | --- | --- | --- | --- | --- |
|  | Kolmogorov-Smirnov^a^ | | | Shapiro-Wilk | | |
|  | Statistic | df | Sig. | Statistic | df | Sig. |
| d2ErrorRate#1 | .327 | 6 | .044 | .830 | 6 | .108 |
| a. Lilliefors Significance Correction | | | | | | |

**Supplementary Table S3.2.** Tests of normality for #2 d2 error rate.

| **Tests of Normality** | | | | | | |
| --- | --- | --- | --- | --- | --- | --- |
|  | Kolmogorov-Smirnov^a^ | | | Shapiro-Wilk | | |
|  | Statistic | df | Sig. | Statistic | df | Sig. |
| d2ErrorRate#2 | .242 | 6 | .200^*^ | .870 | 6 | .227 |
| *. This is a lower bound of the true significance. | | | | | | |
| a. Lilliefors Significance Correction | | | | | | |

| **Descriptives** | | | | |
| --- | --- | --- | --- | --- |
|  | | | Statistic | Std. Error |
| d2ErrorRate#1 | Mean | | 3.5881 | 1.29099 |
|  | 95% Confidence Interval for Mean | Lower Bound | .2695 |  |
|  |  | Upper Bound | 6.9067 |  |
|  | 5% Trimmed Mean | | 3.4585 |  |
|  | Median | | 2.2798 |  |
|  | Variance | | 10.000 |  |
|  | Std. Deviation | | 3.16227 |  |
|  | Minimum | | .91 |  |
|  | Maximum | | 8.60 |  |
|  | Range | | 7.69 |  |
|  | Interquartile Range | | 5.93 |  |
|  | Skewness | | 1.026 | .845 |
|  | Kurtosis | | -.713 | 1.741 |

**Supplementary Table S4.1** d2 Error rate descriptives for #1.

**Supplementary Table S4.2** d2 Error rate descriptives for #2.

| **Descriptives** | | | | |
| --- | --- | --- | --- | --- |
|  | | | Statistic | Std. Error |
| d2ErrorRate#2 | Mean | | 4.4633 | 1.04042 |
|  | 95% Confidence Interval for Mean | Lower Bound | 1.7889 |  |
|  |  | Upper Bound | 7.1378 |  |
|  | 5% Trimmed Mean | | 4.4815 |  |
|  | Median | | 5.1100 |  |
|  | Variance | | 6.495 |  |
|  | Std. Deviation | | 2.54849 |  |
|  | Minimum | | 1.37 |  |
|  | Maximum | | 7.23 |  |
|  | Range | | 5.86 |  |
|  | Interquartile Range | | 5.13 |  |
|  | Skewness | | -.388 | .845 |
|  | Kurtosis | | -2.178 | 1.741 |

**Supplementary Table S5.1** Independent Samples Kruskal-Wallis Test for #1 showing d2 ER between test 1 and 2.

| **Independent-Samples Kruskal-Wallis Test Summary** | |
| --- | --- |
| Total N | 6 |
| Test Statistic | 1.190^a,b^ |
| Degree Of Freedom | 1 |
| Asymptotic Sig.(2-sided test) | .275 |
| a. The test statistic is adjusted for ties. | |
| b. Multiple comparisons are not performed because there are less than three test fields. | |

| **Independent-Samples Kruskal-Wallis Test Summary** | |
| --- | --- |
| Total N | 6 |
| Test Statistic | .429^a,b^ |
| Degree Of Freedom | 1 |
| Asymptotic Sig.(2-sided test) | .513 |
| a. The test statistic is adjusted for ties. | |
| b. Multiple comparisons are not performed because there are less than three test fields. | |

**Supplementary Table S5.2** Independent Samples Kruskal-Wallis Test for #2 showing d2 ER between test 1 and 2.
